# Contrasting macrophage activation by fine and ultrafine titanium dioxide particles is associated with different uptake mechanisms

**DOI:** 10.1186/1743-8977-8-31

**Published:** 2011-10-13

**Authors:** Agnes M Scherbart, Julia Langer, Alexey Bushmelev, Damiёn van Berlo, Petra Haberzettl, Frederik-Jan van Schooten, Annette M Schmidt, Christine R Rose, Roel PF Schins, Catrin Albrecht

**Affiliations:** 1 IUF - Leibniz Research Institute for Environmental Medicine, Düsseldorf, Germany; 2 Institute for Neurobiology, Heinrich-Heine-University Düsseldorf, Germany; 3 Institute of Physical Chemistry, University of Cologne, Cologne, Germany; 4 Department of Health Risk Analysis and Toxicology, Maastricht University, Maastricht, The Netherlands

**Keywords:** NR8383 cells, titanium dioxide, particle internalization, size distribution, agglomeration

## Abstract

Inhalation of (nano)particles may lead to pulmonary inflammation. However, the precise mechanisms of particle uptake and generation of inflammatory mediators by alveolar macrophages (AM) are still poorly understood. The aim of this study was to investigate the interactions between particles and AM and their associated pro-inflammatory effects in relation to particle size and physico-chemical properties.

NR8383 rat lung AM were treated with ultrafine (uf), fine (f) TiO_2 _or fine crystalline silica (DQ12 quartz). Physico-chemical particle properties were investigated by transmission electron microscopy, elemental analysis and thermogravimetry. Aggregation and agglomeration tendency of the particles were determined in assay-specific suspensions by means of dynamic light scattering.

All three particle types were rapidly taken up by AM. DQ12 and ufTiO_2 _, but not fTiO_2 _, caused increased extracellular reactive oxygen species (ROS), heme oxygenase 1 (HO-1) mRNA expression and tumor necrosis factor (TNF)-α release. Inducible nitric oxide synthase (iNOS) mRNA expression was increased most strongly by ufTiO_2 _, while DQ12 exclusively triggered interleukin (IL) 1β release. However, oscillations of intracellular calcium concentration and increased intracellular ROS were observed with all three samples. Uptake inhibition experiments with cytochalasin D, chlorpromazine and a Fcγ receptor II (FcγRII) antibody revealed that the endocytosis of fTiO_2 _by the macrophages involves actin-dependent phagocytosis and macropinocytosis as well as clathrin-coated pit formation, whereas the uptake of ufTiO_2 _was dominated by FcγIIR. The uptake of DQ12 was found to be significantly reduced by all three inhibitors. Our findings suggest that the contrasting AM responses to fTiO_2 _, ufTiO_2 _and DQ12 relate to differences in the involvement of specific uptake mechanisms.

## Introduction

The introduction and application of novel types of nanomaterials and nanodevices is rapidly increasing in recent years. Risks of exposure to nanoparticles (NP, which can be defined as nano-objects with all three external dimensions in the nanoscale, i.e. < 100 nm; [1]) often cannot be reliably estimated at this time. Due to their novel physico-chemical properties, concerns have been raised about their potential to cause adverse effects in biological systems and their impact on human health. Reliable testing strategies to investigate possible health risks caused by nanoparticles (NP) are therefore urgently needed [2,3].

Hints for a potential toxicity of NP arose predominantly from the field of inhalation toxicology, where it has been shown that (aggregates of) specific NP, like carbon black (CB) or titanium dioxide (TiO_2 _), exhibit a markedly higher biological activity at cellular and subcellular levels [2,4,5] when compared to an equal mass dose of their larger sized counterparts [6-8]. Currently, TiO_2 _particles are used widely and in large quantities in many industrial applications like cosmetics, pharmaceuticals, paints and in food industry, as well as in medical and dental prosthesis in either fine (> 100 nm) or ultrafine sizes [9,10].

Animal studies have shown that fTiO_2 _particles predominantly deposit within the deeper regions of the lung and can subsequently induce inflammatory responses [11]. However, this typically does not result in marked lung fibrosis [9,12], unlike other inorganic particles, e.g. crystalline silica [13-15]. Such contrasting outcomes pointed to the existence of fundamental differences between different types of inorganic particles concerning their toxic potential. Investigations of the acute inflammatory effects of an ultrafine and a fine sample of TiO_2 _in rats and mice have shown that the smaller particles are more potent on a mass dose basis, but that the responses do not differ when the samples are adjusted to an equal surface area dose (reviewed in [2]). This suggests that the specific surface area (SSA) of NP *per se *may define their pro-inflammatory effects. However, on the cellular level biological effects of NP are considered to be driven by their specific physico-chemical interactions with cells and subcellular constituents, including initial recognition and/or interference with specific membrane associated receptors [16]. This specific particle-cell-interaction may also explain observations in other studies where associations between the SSA and specific toxic effects were not as clear (e.g. [17]).

Alveolar macrophages (AM) are professional phagocytes accounting for approximately 95% of airspace leukocytes in the healthy lung, which generally represent the first cell type that gets into contact with inhaled pathogens [18]. The AM cell line NR8383 has been extensively characterized and is widely accepted as a reliable surrogate for freshly obtained primary AM [19]. In a previous study, we demonstrated the participation of the classical phagocytosis Fcγ receptor II (FcγRII) in the uptake of fine crystalline silica particles (with a mean geometric diameter of about 1 μm) by NR8383 AM [20]. Other studies have shown an association between FcγRII stimulation by interferon γ in the monocyte cell line U937 as well as in primary human blood monocytes, and the induction of a signal cascade which is connected to phospholipase (PLC)γ-1. Activation of PLCγ-1 is known to increase the concentration of intracellular calcium ([Ca^2+ ^]_i _) which in turn can activate nicotinamide adenine dinucleotide phosphate (NADPH) oxidases and thus the generation of ROS via the so-called oxidative burst [21,22]. Participation of other membrane receptors including the class A scavenger receptor (SR-A) and the macrophage receptor with collagenous structure (MARCO) has also been described to be of importance for the uptake of fine-sized TiO_2 _and silica particles, but not for the internalization of carbonaceous particles [23,24]. Taken together, these observations provide strong evidence that particle type-specific mechanisms of uptake exist in macrophages. However, the exact route(s) by which NP can enter these cells and their impact on subsequent cellular responses are still poorly understood. Elucidation of these mechanisms will provide an important step for the risk assessment of NP and for potential medical and pharmaceutical applications of newly engineered NP.

Interaction of AM with respirable particles can lead to the production of ROS and secretion of a large variety of cytokines, chemokines and other, typically pro-inflammatory mediators. These include TNF-α and interleukin (IL)-1β, both early pro-inflammatory cytokines which in turn are capable to activate various secondary mediators and as such orchestrate the recruitment of further immune cells, like neutrophilic granulocytes [25]. Many of these cytokines and chemokines are regulated by redox-sensitive transcription factors like nuclear factor kappa B (NF-κB) and/or activator protein 1 (AP-1), which in turn are regulated by second messengers including calcium and ROS [26,27]. Enhanced [Ca^2+^]_i _levels are known to lead to the activation of protein kinase C (PKC) which is involved in the activation of NF-κB [28,29]. The involvement of [Ca^2+^]_i _in the pro-inflammatory responses of AM has recently been established for fine crystalline silica particles (54) as well as for carbonaceous NP, i.e. ultrafine carbon black [27].

In our current study, two types of TiO_2 _with different size distributions were investigated, i.e. fTiO_2 _and ufTiO_2 _. The aims of our study were to analyze (i) differences in uptake mechanisms for these samples in AM, and (ii) how the uptake associates with various cellular responses in AM that are considered to play a role in the adverse health effects of inhaled particles. The established inflammogenic and fibrogenic crystalline silica sample DQ12 was used as reference particle [15,30-33]. Intra- and extracellular responses of AM were investigated via the analysis of particle uptake, cytotoxicity, changes in [Ca^2+^]_i _, ROS generation as well as the induction of various markers of inflammation and oxidative stress, i.e. NF-κB, TNF-α, IL-1β, inducible nitric oxide synthase (iNOS) and heme oxygenase-1 (HO-1). Particle type-specificity of internalization by AM was investigated by uptake analysis in the absence or presence of specific inhibitors, i.e. cytochalasin D (CytD), chlorpromazine (Chl), filipin III, FcγRII antibody as well as by evaluation of uptake at 4 versus 37°C. Since former studies have shown that TiO_2 _particles tend to reside as aggregates as well as to form larger agglomerates in suspension depending on the type of buffers [34], in the present study special emphasis was also put on the characterization of the specific particle suspensions used for the various biological tests. Elemental analysis (EA) and thermogravimetric analysis (TGA) have been employed to exclude the presence of organic residues in the investigated powders that could impact on AM responses. Morphology and dispersion behavior of the samples in the different media was evaluated by means of transmission electron microscopy (TEM), dynamic light scattering (DLS) and dark field light scattering microscopy (DF-LSM).

## Materials and methods

### Particle samples used

Three types of particles were used in this study, i.e. ufTiO_2 _and fTiO_2 _and a respirable quartz sample (DQ12). fTiO_2 _was obtained from Sigma-Aldrich and is a pure anatase sample with a reported mean diameter of about 250 nm [8]. The ufTiO_2 _sample originates from Degussa (Hanau, Germany) and represents a mixture of 80% anatase and 20% rutile with a reported mean particle size of 25 nm [8]. The DQ12 sample originates from Dörentrup, Germany (IUF batch 6) and represents a highly pure quartz (99.1%) with a mean particle diameter of 960 nm [32]. The specific surface areas of the samples measured according to the method of Brunauer, Emmert and Teller [35] are 50 m^2^/g, 10 m^2^/g and 9.6 m^2^/g, for ufTiO_2 _, fTiO_2 _and DQ12, respectively.

### Particle characterization

In order to obtain additional information on particle composition and size, the batches were subjected to TGA, EA, DLS, DF-LSM and TEM. EA of the powders was carried out with a Perkin-Elmer Analyzer 2400 with an accuracy of measurement of 0.3%. TGA experiments were performed on powder samples on a Netsch STA 449 C Jupiter at a constant heating rate of 10 K min^-1 ^in an argon atmosphere between 30 and 600°C. The solid residues at 600°C are attributed to the inorganic component. TEM images were taken using a Philips EM 208 S. DLS measurements were performed on a High-Performance Particle Sizer HPP5002 (Malvern Instruments) after their suspension in water, Hank's buffered saline solution (HBSS^(+/+)^, phenol red free, with Mg^2+ ^and Ca^2+^; Invitrogen GmbH, Karlsruhe, Germany) and FCS-containing cell culture medium at 25°C, using 1 × 1 cm^2 ^polystyrene cuvettes. Particle size distributions were derived from a deconvolution of the measured intensity autocorrelation function by the non-negative least-squares algorithm included in the DTS software. The suspensions used for this analysis were prepared in the same way as those used for the biological testing as described below. DLS analysis of the ufTiO_2 _particle suspension was also performed after filtration through a 450 nm membrane filter to further evaluate the influence of aggregate/agglomerate formation on this method.

### Preparation of the particle suspensions and cell treatments

NR8383 rat AM (ATCC, Manassas, USA) were cultured in Kaighn's modified medium (F12-K Nutrient Mixture, Gibco, Eggenstein, Germany) containing 15% FCS, 1% penicillin/streptomycin and 1% glutamine (all purchased from Sigma-Aldrich, Taufkirchen, Germany) and incubated in a humidified incubator (Heraeus, BB 6060 CU) at 37°C and 5% CO_2 _. Three days before each experiment, cells were seeded in a concentration of 1.25 × 10^5 ^cells/cm^2 ^in the indicated culture dishes. If not otherwise mentioned, incubations took place at 37°C and 5% CO_2 _.

Particles were heated at 220°C for 16 h in order to destroy potential endotoxins, which are known to be potent activators of AM. Immediately before the experiment, particles were freshly suspended either in complete cell culture medium (see above) for most of the experiments except for ROS and calcium measurements, for which suspension was performed in HBSS^(+/+) ^(phenol red free, with Mg^2+ ^and Ca^2+^) or saline, respectively. The evaluation of mRNA expression changes was done under both treatment conditions, i.e. using particles suspended in either HBSS^(+/+) ^or complete medium. All suspensions were sonicated in a water bath for 10 min (Sonorex TK 52, Schaltech, Mörfelden-Walldorf, Germany) immediately prior to addition to the cells. Particles were added to the AM at concentrations of 10, 20, 40, 80 μg/cm^2 ^for 1, 4 or 24 h as indicated.

### Measurement of particle uptake by flow cytometry

The uptake of particles by the AM was analyzed via flow cytometry. Measurement was performed with a FACS Calibur (Becton Dickinson, Heidelberg, Germany). The sideward scatter (SSC) which is directly related to cell granularity was used as a marker of particle uptake [36], whereas the forward scatter (FSC) mainly correlates to the cell size.

For inhibition experiments, cells were preincubated for 30 min with the following substances: CytD (1.5 μg/mL; Sigma, Taufkirchen, Germany) to inhibit actin recruitment, Chl (5 μg/mL; Sigma-Aldrich, Taufkirchen, Germany) to disable the formation of clathrin coated pits (CCP), and an antibody against the phagocytotic FcγRII (CD32, 5 μg/mL; BD Biosciences, Heidelberg, Germany) to avoid specific receptor binding. Dimethyl sulphoxide (DMSO, 0.1%; Sigma, Taufkirchen, Germany) was applied as vehicle control for CytD. The IgG1κ monoclonal antibody was used as isotype control (5 μg/mL; BD Biosciences, Heidelberg, Germany) for the FcγRII antibody experiments. Cells were treated with particles at concentrations of 10, 20 or 40 μg/cm^2 ^for 1 or 3 h. NR8383 cells were gently scraped from the culture dishes on ice, centrifuged (200 × g, 10 min, 4°C), washed with 300 μl of ice cold HBSS^(-/-) ^and centrifuged again. The pellet was resuspended in 200 μl ice cold HBSS^(-/-)^. In total 15,000 events were counted. For calculation cell debris and free particles were excluded by an electronical gate containing AM of all sizes and granularities in a FSC-SSC-histogram. Univariant histograms of SSC determined the median of cell granularity used as measure of particle uptake by AM. Data were detected with CellQuest 3.3 and analyzed using CellQuest Pro (Becton Dickinson, Heidelberg, Germany).

### Microscopical evaluation of uptake

In order to microscopically investigate AM after a 24 h treatment with particles cytospin slides were prepared. Therefore, NR8383 cells were scraped, centrifuged (200 × g, 5 min, 4°C), washed and resuspended in sterile, ice cold phosphate buffered saline (PBS). Then 2 × 10^5 ^cells were spun onto glass slides (600 rpm, 5 min) using a Cytospin3 (Shandon GmbH, Frankfurt, Germany). After drying and May-Grünwald-Giemsa-staining (Merck, Darmstadt, Germany) preparations were analyzed via light microscopy (Olympus BX60, Hamburg, Germany).

### Cytotoxicity

Effects of particles on cell viability were determined using the WST-1 assay (Roche Diagnostics GmbH, Mannheim, Germany) which is based on the principle of the reduction of the stable tetrazolium salt WST-1 to a soluble violet formazan product within the mitochondria of viable cells. For this assay, NR8383 cells were seeded in octoplicate in 96-well microtiter plates. After 24 h of particle treatment, 10 μL WST-1 solution (Roche Diagnostics GmbH, Mannheim, Germany) was added to 5 wells per treatment or control and incubated for further 2 h. The other three wells were used as controls for the absorption by the particles and therefore measured without WST-1 substrate application. Optical density was detected at 450 nm using the Multiskan ELISA reader (Thermo Fisher Scientific, Dreieich, Germany). For data calculation, the mean of the obtained values of the wells without WST-1 was subtracted from the mean of the WST-1 substrate treated samples and expressed as percentage of control cells. To investigate potential reagent binding to particles, which could lead to a false interpretation of toxicity [3], cell free experiments were performed in WST-1- as well as formazan-containing suspensions spiked with particles. Such artifacts could be excluded for the particles used in our present study.

### Calcium imaging

In order to investigate the relation between particle exposure and intracellular calcium, wide-field fluorescent imaging was employed to measure changes in [Ca^2+^]_i _in individual cultured AM upon particle treatment. NR8383 cells were seeded onto sterile coverslips coated with poly-D-lysine hydrobromide (Sigma-Aldrich, Taufkirchen, Germany) and used for experiments after 3 - 4 days. Loading with the Ca^2+ ^sensitive fluorescent dye Fura-2 and fluorescence measurements were performed in saline containing (in mM): NaCl 125, KCl 3, NaH_2 _PO_4 _1.25, MgSO_4 _2, CaCl_2 _2, HEPES 25, D-glucose 10 (pH 7.4). The acetoxymethyl ester form of the fluorophore was dissolved as 5 mM stock solution in 20% Pluronic acid in DMSO and stored at -20°C. For dye loading, coverslips were incubated for 90 min at room temperature in saline containing 0.5 mM Fura-2- acetoxymethyl ester. Following loading, cells were kept in dye-free saline for at least 30 min to ensure de-esterification of the dye before starting the imaging experiments.

Conventional wide-field fluorescent imaging was performed employing an imaging system (Till Photonics GmbH, Munich, Germany) in conjunction with an upright microscope (Axioskop, Zeiss, Oberkochen, Germany) equipped with a cooled CCD camera (SensiCam QE, PCO, Kelheim, Germany). Cells loaded with Fura-2 were excited every 5 s using a monochromator (polychrome V, Till Photonics GmbH); fluorescence emission from regions of interest (ROIs) placed around AM somata was detected by the camera. Emission intensities > 440 nm were collected after alternate excitation at 357 and 380 nm, and background-corrected fluorescent ratios (F357/380) were calculated. Background fluorescence was determined from coverslip areas devoid of cellular material.

Baseline [Ca^2+^]_i _was recorded under control conditions and for at least 70 min during exposure to either ufTiO_2 _or fTiO_2 _particles at concentrations of 10 or 20 μg/cm^2^. To quantitatively analyze and compare calcium fluctuations in response to different particles, integrals for ratio values for specific 10 min time windows (one for control experiments; 15 - 25 min and 55 - 65 min after particle application) were calculated for each individual cell employing OriginPro Software (OriginLab Corporation, Northampton, MA).

### Intracellular ROS measured by DCFH-DA

To quantify intracellular ROS the cell-permeable non-fluorescent probe 2',7'-dichlorodihydrofluorescein diacetate (DCFH-DA; Sigma, Saint-Louis, Missouri, USA) was used. After entering the cell, DCFH-DA loses its diacetate group by the action of esterases. The oxidation of this probe leads to the highly fluorescent DCF. Prior to particle treatment, NR8383 cells seeded in 96-well plates were washed and replaced by HBSS^(+/+) ^to avoid scavenging effects of DCFH-DA by medium components. After a recovery time of 30 min, AM were pre-incubated with 100 μM DCFH-DA for another 20 min in the incubator. Afterwards cells were washed and allowed to recover for another 30 min before they were treated with 40 μg/cm^2 ^of particles. The change in DCFH-DA fluorescence over time was detected via fluorescence reader (Synergy2, BioTek Instruments Inc., Bad Reichenhall, Germany) for 3 h at 37°C.

### Measurement of extracellular ROS by Electron Paramagnetic Resonance (EPR) spectroscopy with spin trapping

For the analysis of extracellular ROS, NR8383 cells were seeded in 96 well plates. Cells were washed and the medium was replaced by HBSS^(+/+) ^followed by a recovery time of 30 min. AM were treated with particles at concentrations of 10 or 40 μg/cm^2^. The spin trapping agent 5,5-dimethyl-1-pyrroline-N-oxide (DMPO, 0.11 M, Sigma-Aldrich, Taufkirchen, Germany) was added simultaneously with the particles; cells were incubated for 3 h. Cell-free supernatants were harvested and immediately measured for radical formation using a MiniScope MS200 Spectrometer (Magnettech, Berlin, Germany) with the following instrumental settings: room temperature, microwave frequency = 9.39 GHz, magnetic field = 3360 G, sweep width = 100 G, scan time = 30 s, number of scans = 3, modulation amplitude = 2 G, receiver gain = 900. Quantification was carried out on first derivation of EPR signal of the characteristic DMPO-OH quartet, as the mean of amplitudes, and outcomes are expressed in arbitrary units (a.u.). DQ12 and the well-known PKC activator phorbol 12-myristate 13-acetate (PMA) were used as positive controls.

### Immunocytochemistry (IHC) for NF-κB

Transcriptional activation of the nuclear factor κB was microscopically analyzed by the nuclear translocation of the p65 subunit. Therefore, cells were seeded into 4-chamber slides and treated with 40 μg/cm^2 ^of particles. After 1 h, NR8383 cells were fixed (4% paraformaldehyde/PBS, pH 7.4) and permeabilized (0.1% TritonX-100, 5 min). Unspecific binding sites were blocked by goat normal serum followed by an overnight incubation with anti-NF-κB (p65) antibody (1:500, Santa Cruz Biotechnology, CA, USA). Slides were incubated with secondary antibody Alexa-488 (1:200, Molecular Probes, OR, USA) for 1 h before mounting the cover slip with Ultra Cruz Mounting Medium containing DAPI (Santa Cruz Biotechnology, CA, USA). Fluorescence images were taken with the Axio Observer.D1 fluorescence microscope (Carl Zeiss MicroImaging GmbH, Göttingen, Germany).

### TNF-α and IL-1β release

NR8383 cells were seeded onto 24-well microtiter plates. After 24 h of particle treatment, cell-free supernatant was collected, centrifuged (200 × g, 10 min, 4°C) and aliquots were stored at -20°C. Supernatants were analyzed using a TNF-α or IL-1β ELISA kit (R&D Systems, Wiesbaden, Germany) according to the manufacturer's manual and using a Multiskan ELISA reader (Thermo Fisher Scientific, Dreieich, Germany).

### Quantitative RT-PCR analysis of gene expression

NR8383 cells were seeded in 6-well plates, treated with particles for 4 h, scraped and centrifuged (200 × g, 5 min, 4°C). The pellet was resuspended in 0.5 ml Trizol^® ^Reagent (Invitrogen GmbH, Karlsruhe, Germany) and stored at -20°C until further use. The RNeasy^® ^mini kit (Qiagen, Hilden, Germany) coupled to DNAse treatment was used to purify total RNA from salts and residual DNA. Quantity and purity of RNA were evaluated using spectrophotometry at 230, 260, 280, and 320 nm. cDNA was synthesized using the iScript™ cDNA Synthesis kit (BioRad, CA, USA), starting from 0.5 μg of RNA. cDNA was diluted 15 × in RNAse-free water before use. PCR primers for rat HO-1, iNOS and the housekeeping gene GAPDH were designed using Primer Express software (Applied Biosystems). Primer sequences for HO-1 were 5'-GGG AAG GCC TGG CTT TTTT -3' (forward) and 5'-CAC GAT AGA GCT GTT TGA ACT TGGT -3' (reverse), for iNOS 5'-AGG AGA GAG ATC CGG TTC ACA GT-3' (forward) and 5'-ACC TTC CGC ATT AGC ACA GAA-3' (reverse) and for GAPDH 5'-TGA TTC TAC CCA CGG CAA GTT-3' (forward) and 5'-TGA TGG GTT TCC CAT TGA TGA-3' (reverse). qRT-PCR was performed with a MyiQ Single Color real time PCR detection system (BioRad) using iQ™ SYBR^® ^Green Supermix (Biorad), 5 μL diluted cDNA, and 2.5 μL of 0.3 μM forward and reverse primer in a total volume of 25 μL. PCR was conducted as follows: a denaturation step at 95°C for 3 min was followed by 40 cycles at 95°C (15 s) and 60°C (45 s). After PCR, a melt curve (60 - 95°C) was generated for product identification and purity. PCR efficiency of all four primer sets, as assessed by the use of cDNA dilution curves, was 90 - 100%. Data were analyzed using the MyiQ Software system (BioRad) and were expressed as relative gene expression (fold increase) using the 2-^ΔΔCt ^method [37].

### Statistical Analysis

All biological assays were performed in at least three independent experiments. Data are presented as mean ± SEM unless indicated otherwise. Statistical analysis was performed using SPSS 18.0 for Windows using analysis of variance (ANOVA) with Dunnett or LSD post hoc comparison as indicated for the specific data. Differences compared to untreated control cells were considered significant at * p < 0.05, ** p < 0.01 and *** p < 0.001. Differences in inhibition experiments compared to the appropriate particle treatment were indicated as # p < 0.05, ## p < 0.01 and ### p < 0.001.

## Results

### Particle sample characteristics

**Figure 1:**
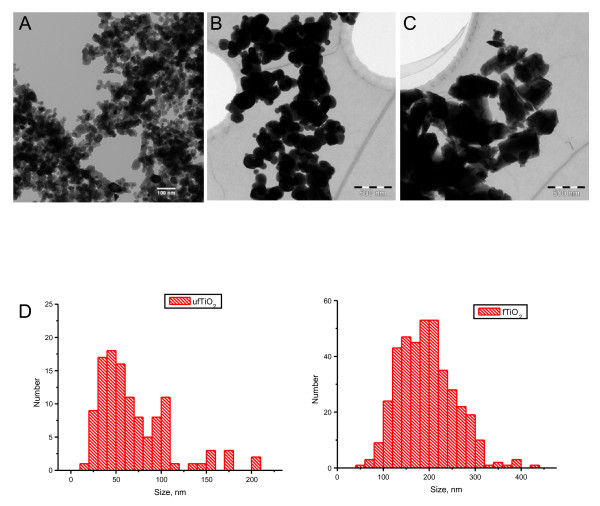
**TEM images and size distribution of particles**. Particles were suspended and prepared for TEM measurements in deionized water. (A) ufTiO_2 _and (B) fTiO_2 _are regular and spherical particles in contrast to (C) crystalline silica DQ12 particles that display a very irregular shape. (D) Particle size histograms are derived from the appropriate TEM analysis for primary particles of ufTiO_2 _and fTiO_2 _.

In order to verify the absence of organic residues in the particle samples, we performed EA and TGA in the powders. In the former, no carbon content was detected within the experimental error, and in the latter, no significant weight loss was found attributable to organic combustible contents, up to a temperature of 600°C (data not shown). The morphology of the samples was investigated by means of TEM. Representative images (Figure 1) showed for the TiO_2 _samples individual particles of nearly spherical shape and moderate size distribution. From the images, particle size histograms were extracted (see Figure 1D), revealing a number-average primary particle diameter of 69.1 nm (± 39.9 nm SD) for ufTiO_2 _particles and of 194.9 nm (± 60.8 nm SD) for fTiO_2 _, respectively. Figure 1C shows a representative image of the control sample DQ12. A detailed characterization of this sample has been performed in a previous study, revealing a mean diameter of 960 nm [32].

### Evaluation of particle suspensions used for biological testing

It is well-known that for (nano)particle dispersions the actual object size can differ significantly from the size of the primary particles and their aggregates due to agglomeration processes. Therefore, we compared the core size of the primary particles obtained from TEM with results from dispersion-based methods. For this purpose, DLS and DF-LSM experiments were carried out. Both methods are based on the analysis of the distribution of the diffusion coefficient measured from scattered light signals. While in DLS the signal fluctuations are used directly to obtain information on the autocorrelation function, in DF-LSM the signals are used to track individual objects and analyze their Brownian diffusion perpendicular to the laser direction. For ufTiO_2 _as well as fTiO_2 _, particle dynamics as examined by DLS were in accordance with the presence of predominantly primary particles or small aggregates in water dispersion after ultrasonification and partly filtration by a 450 nm microfilter. This is evident from the high correlation of the number-average hydrodynamic particle diameter as extracted by DLS (see Table 1) with the average core diameter as observed by TEM. Similar values were also obtained for the cell culture-based particle dispersions. In the latter, after filtration which was performed to investigate the impact of aggregate/agglomerate formation on the DLS measurements, the scattering signal was dominated by small colloids (~ 6 nm). This signal could be attributed to proteins which are abundant in the FSC containing medium. The proteins contained within the FCS stabilize the particles in the dispersion and hence prevent their sedimentation. In the HBSS-based suspensions, however, the DLS measurements indicated the presence of agglomerates with a diameter of up to the micrometer range (see Table 1, value in brackets). The findings are in accordance with the observation of a lower sedimentation stability of these buffer-based samples compared to water- or complete culture medium-based dispersions.

**Table 1:** Characteristics of particle dispersions as measured by DLS

sample	Dispersant	*d*_h _^a^	PDI^b^
	water	55.4^c^ (891.2)	0.22
	
**ufTiO_2 _**	cell culture medium	57.5	0.33
	
	HBSS	164.2^c^ (2018)	0.26

	Water	321.2	0.18
	
**fTiO_2 _**	cell culture medium	448.6	0.16
	
	HBSS	936.6	0.64

^a ^number-average hydrodynamic diameter [nm]

^b ^polydispersity index

^c ^sample was filtered (450 nm microfilter) prior to experiment

in brackets: results of the comparable measurement of unfiltered sample

Importantly, for the ufTiO_2 _particles the mass percentage of the filtered fraction was 20% of the total mass (data not shown). The large fraction of the non-filtered material at least in part accounts for larger agglomerates which may be inappropriately measured by the DLS method. As such, the data of the unfiltered samples have to be interpreted with caution. Nevertheless, the measurements of the unfiltered suspensions (Table 1) showed that the ultrafines tended to from larger agglomerates than their fine counterparts. These findings were also supported by data obtained from DF-LSM (not shown), and are important in view of the cell based assays.

### Dose-dependent particle uptake in alveolar macrophages

**Figure 2:**
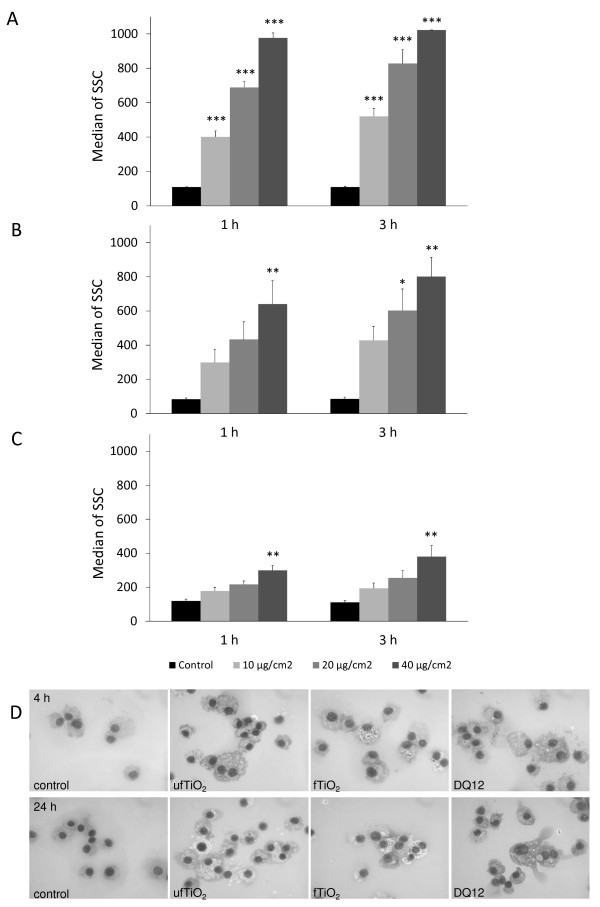
**Concentration- and time-dependent particle uptake by NR8383 cells**. Results of FACS analysis demonstrate increased particle uptake by AM based on SSC of laser light indicating cellular granularity. AM were treated with (A) ufTiO_2 _, (B) fTiO_2 _and (C) DQ12 particles in concentrations of 10, 20 or 40 μg/cm^2 ^for 1 or 3 h. (D) Particle internalization by NR8383 cells are demonstrated in MGG-stained cytospin preparations. Light microscopic images show AM either untreated or treated with 10 μg/cm^2 ^of particles for 4 h (upper panel) or 24 h (lower panel). Original magnification 1000-fold (Olympus BX60). Figure A - C represent median ± SEM of three independent experiments, with * p < 0.05, ** p < 0.01 and *** p < 0.001 vs. control (ANOVA with Dunnett post-hoc comparison).

The uptake of particles by NR8383 AM was determined by measuring the granularity of the cells via flow cytometry. These measurements revealed a dose-dependent uptake (10, 20 or 40 μg/cm^2^) of all tested particles after 1 as well as 3 h (Figure 2, panels A-C). Comparison of the SSC results also indicates that the smaller the ingested particles, the higher is the light scattering caused by the elevated granularity of the cells. This was confirmed by comparison of cell free particle suspensions revealing a median SSC of 885, 728 or 228 for ufTiO_2 _, fTiO_2 _or DQ12, respectively. Uptake was also verified by light microscopy of treated NR8383 cells (Figure 2D).

### Cell toxicity following particle exposure

**Figure 3:**
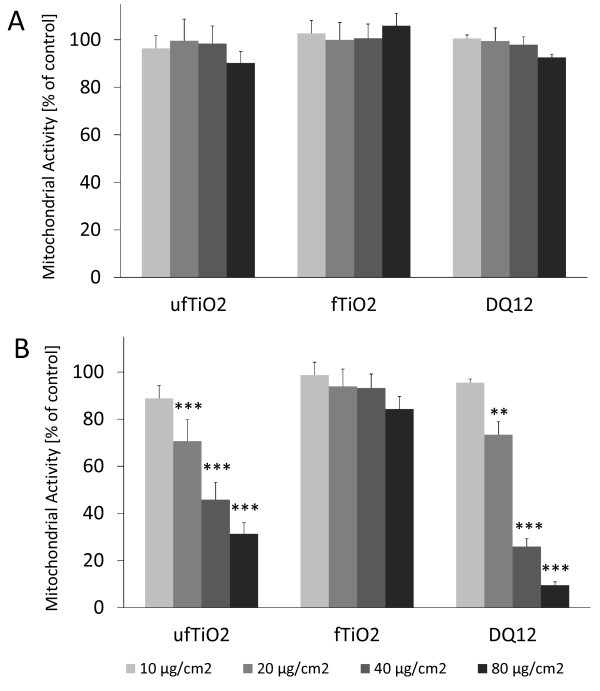
**Particle-dependent effects on cell viability in NR8383 cells**. Mitochondrial activity of particle-treated AM shows (A) no impairment of cell viability after 4 h. (B) Particle treatment of 24 h reveals toxicity for ufTiO_2 _and DQ12 particles at concentrations of 20 μg/cm^2 ^and above, whereas fTiO_2 _does not result in toxicity up to 80 μg/cm^2^. Figures represent mean ± SEM of three independent experiments, with ** p < 0.01 and *** p < 0.001 vs. control (ANOVA with LSD post-hoc comparison).

Viability of AM after 4 and 24 h of particle treatment was determined by measurement of mitochondrial dehydrogenase activity. This investigation showed no cytotoxic effects after 4 h for all three tested particles (Figure 3A) but marked differences between fTiO_2 _and ufTiO_2 _particles after 24 h (Figure 3B). Treatment of the AM with ufTiO_2 _particles already caused toxic responses at a concentration of 20 μg/cm^2 ^which was comparable to the responses of the positive control DQ12. For fTiO_2 _particles no effects on cell viability were found up to the highest tested concentration of 80 μg/cm^2^.

### Ultrafine and fine TiO_2 _exposure induce intracellular increase of calcium

**Figure 4:**
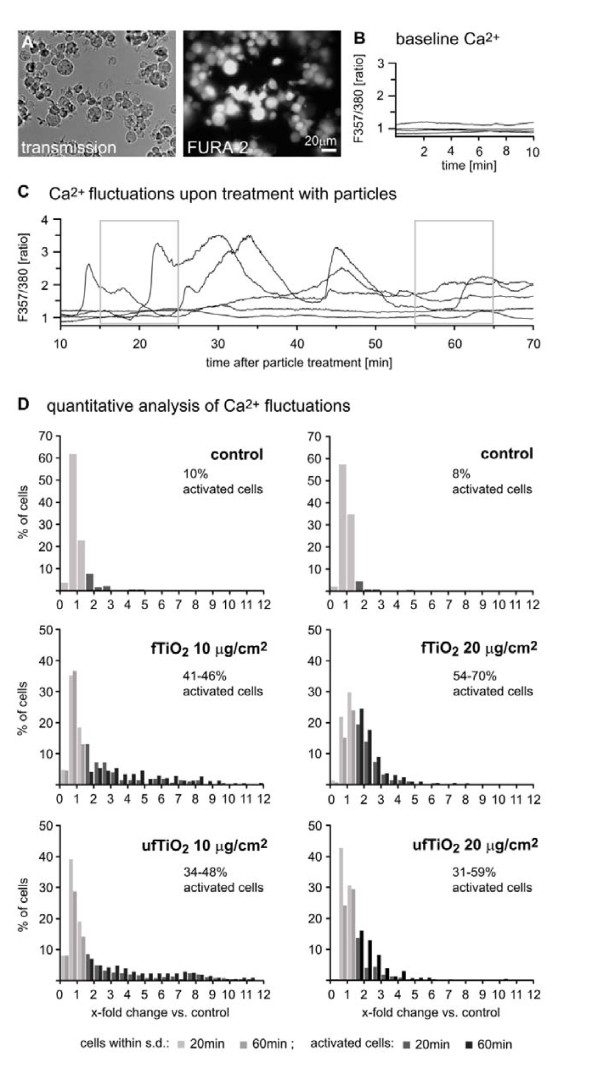
**Increase of [Ca^2+^]_i _after exposure of NR8383 cells to TiO_2 _samples**. (A) Transmission image of AM (left) and image of Fura-2 fluorescence (excitation at 357 nm) (right). (B) Control experiments showing Fura-2 fluorescence ratio of untreated AM. (C) Calcium fluctuations in individual cells in response to particle application (ufTiO_2 _20 μg/cm^2^) over time. Some cells exhibit large calcium fluctuations, while others do not respond. Grey boxes indicate the time windows for which quantitative analysis shown in D was performed. (D) Quantitative analysis of calcium fluctuations: The integral of ratio traces was calculated for 10 min time windows for each individual cell. The resulting integral values were normalized to the mean of control values obtained in the absence of particles. Histograms show the percentage of cells exhibiting a 1.5 to 12 fold increase in calcium ratio integrals relative to controls. Light gray columns represent integral values within the standard deviation of controls. Dark columns represent signals of activated cells. Data are from 57 experiments on 39 coverslips, number of cells for each group: n = 198 - 443.

Calcium is an important second messenger involved in a multitude of intracellular signaling pathways. We, therefore, investigated the impact of exposure to TiO_2 _particles on the calcium concentration ([Ca^2+^]_i _) of individual AM by performing ratiometric imaging with Fura-2 (Figure 4A). Under control conditions with no added particles, the baseline calcium concentration was stable (Figure 4B). Addition of either ufTiO_2 _or fTiO_2 _caused an increase in the intracellular calcium concentration in a large number of cells in the field of view, some cells responded with large and random calcium oscillations (Figure 4C).

To quantitatively analyze and compare calcium fluctuations in response to different particles and different concentrations, integrals for ratio values for specific 10 min time windows (one for control experiments; 15 - 25 min and 55 - 65 min after particle application) were calculated for each individual cell (Figure 4D). The resulting integral values were normalized to the mean of control values (obtained in the absence of particles). Cells, displaying values not covered by the standard deviation of control, were classified as "activated". A small number of such activated cells (8 - 10%) were already found in the control (Figure 4D, upper histograms). After addition of particles, however, the number of activated cells increased to 30 - 70% (Figure 4D). No consistent differences in percentage of activated cells nor amplitude of calcium fluctuations were found between ufTiO_2 _and fTiO_2. _

### Particle-induced intracellular ROS generation

**Figure 5:**
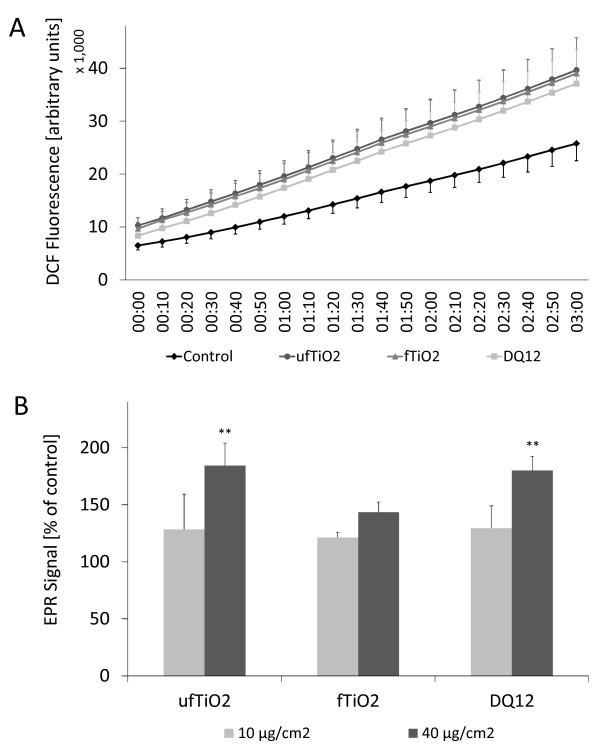
**ROS generation by NR8383 cells after particle treatment**. (A) Intracellular ROS were measured via fluorescence of DCFH-DA after particle treatment of 40 μg/cm^2^. (B) EPR measurements in NR8383 supernatant using the spintrap DMPO show a concentration-dependent generation of ROS induced by treatment with both TiO_2 _and DQ12 particles. In both experiments AM were treated for 3 h. Figures represent mean ± SEM of four independent experiments, with * p < 0.05 and ** p < 0.01 vs. untreated control (ANOVA with LSD post-hoc comparison) in Figure B.

To study the ability of ufTiO_2 _, fTiO_2 _and DQ12 particles to cause ROS generation in the NR8383 cells, measurements were conducted over a period of 3 h. Formation of the fluorescent DCF, representing intracellular ROS formation within the particle treated AM is shown in Figure 5A. Intracellularly generated ROS were clearly observed upon particle treatment compared to untreated NR8383 cells. No significant differences were found between the different types of particles.

### Particle-induced extracellular ROS generation

Extracellular ROS were detected upon 3 h of particle treatment in the supernatant of NR8383 cells by EPR coupled to spin trapping with DMPO. Results are shown in Figure 5B. A clear dose dependency was observed for all three particle types, with effects reaching statistical significance for ufTiO_2 _and DQ12 at the higher concentration of 40 μg/cm^2^.

### Induction of markers of oxidative stress and inflammation

**Figure 6:**
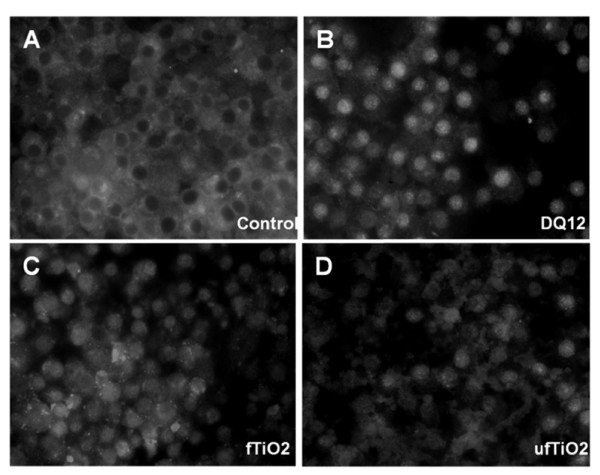
**Induction of NFκB signaling after particle treatment**. NR8383 cells were treated with particles as indicated in concentrations of 40 μg/cm^2 ^for 1 h. Compared to (A) untreated AM, (B) DQ12-treated cells show the strongest nuclear staining, while (C) fTiO_2 _and (D) ufTiO_2 _particles demonstrate a lower nuclear staining. Original magnification: 400-fold (Zeiss Axio Observer.D1).

ROS as well as [Ca^2+^]_i _are known to play an important role in activating several signaling pathways such as MAP kinases and redox-sensitive transcription factors including NF-κB, which can lead to the production of pro-inflammatory molecules and mediators. Therefore, we investigated the effects of the different particles on NF-κB activation, release of TNF-α and IL-1β as well as changes in mRNA expression of HO-1 and iNOS in NR8383 cells. In non-activated cells, NF-κB-specific fluorescence, reported by an antibody against RelA(p65), was located in the cytoplasm of the AM as shown in Figure 6A. A distinct increase in nuclear fluorescence staining, indicating activation of the NF-κB pathway, was seen after treatment with the positive control DQ12 (Figure 6B). Treatment of NR8383 cells with ufTiO_2 _(Figure 6D) was found to cause an increase in the nuclear p65 staining albeit less strong than that following DQ12 treatment. Treatment with fTiO_2 _showed a weak signal (Figure 6C).

**Figure 7:**
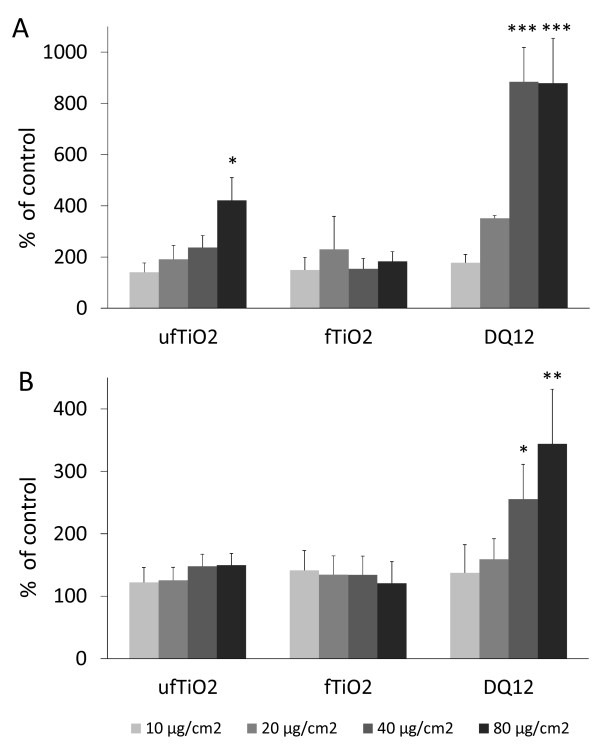
**Particle-dependent release of TNF-α and IL-1β**. (A) ufTiO_2 _and DQ12 particles trigger the release of TNF-α by AM in a concentration- dependent manner. (B) Only DQ12 particles cause the release of IL-1β by AM at the highest concentrations. Data are presented as mean ± SEM of three independent experiments, with * p < 0.05, ** p < 0.01 and *** p < 0.001 vs. medium control (ANOVA with LSD post-hoc comparison).

The ability of the different particle types to induce TNF-α and IL-1β release from NR8383 cells is shown in Figure 7. TNF-α release was found to be induced by ufTiO_2 _particles in a concentration-dependent manner, but not by fTiO_2 _particles (Figure 7A). DQ12 was the most potent particle type, showing a significantly increased TNF-α release at 40 μg/cm^2^, whereas a significant effect for ufTiO_2 _was only found at the highest concentration tested (80 μg/cm^2^). In contrast to the observations for TNF-α, the release of IL-1β from NR8383 cells was only increased after treatment with DQ12 particles (Figure 7B). Neither ufTiO_2 _nor fTiO_2 _were capable of initiating an increased IL-1β release from NR8383 cells at the concentrations tested.

**Figure 8:**
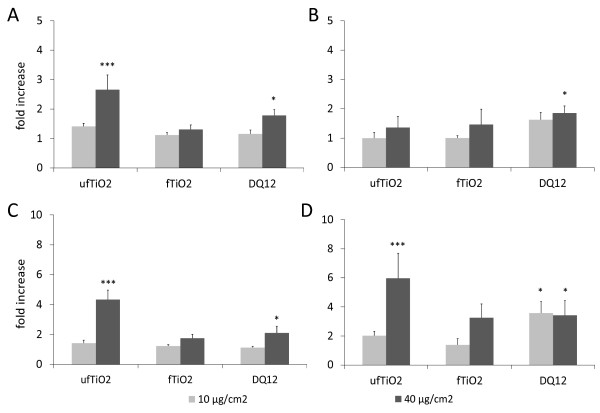
**Particle-dependent impact on the mRNA regulation of HO-1 and iNOS**. NR8383 cells were treated with 10 or 40 μg/cm^2 ^of the indicated particles for 4 h in complete culture medium (A and C) or HBSS (B and D). In medium (A), ufTiO_2 _and DQ12 trigger mRNA upregulation of the stress-response gene HO-1 in a concentration-dependent manner, whereas in HBSS (B) a significant induction was only observed with DQ12. The synthesis of the inflammatory marker iNOS is induced on the mRNA level by both ufTiO_2 _and DQ12 at a concentration of 40 μg/cm^2 ^in medium (C) as well as in HBSS (D). In HBSS, DQ12 also elicited significant induction of iNOS at the lower treatment concentration. All data are corrected for GAPDH. Figures represent mean ± SEM of seven (A, C) or three (B, D) independent experiments, with * p < 0.05, ** p < 0.01 and *** p < 0.001 vs. medium control (ANOVA with LSD post-hoc comparison).

Results of qRT-PCR analyses of the stress response gene HO-1 and the inflammatory gene iNOS are shown in Figure 8 after treatment of the cells in complete culture medium (8A and 8C) or in HBSS (8B and 8D), respectively. Under full medium conditions, a significant higher HO-1 and iNOS mRNA expression was observed after 4 h incubation with 40 μg/cm^2 ^ufTiO_2 _as well as DQ12. When treated with HBSS suspensions, the ability of the ufTiO_2 _to induce HO-1 mRNA expression was markedly impaired (8A versus 8B), whereas its effect on iNOS remained significant (8C versus 8D). In contrast, DQ12 in HBSS was able to induce the expression of both genes (B and D). The fTiO_2 _particles showed no notable effect on the mRNA expression of either gene.

### Determination of specific internalization routes for the investigated particles

**Figure 9:**
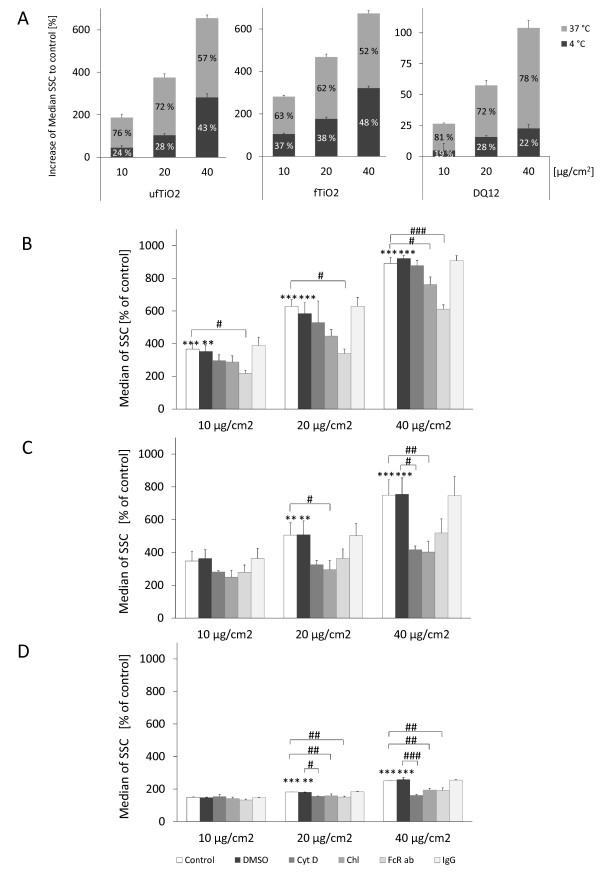
**Particle-specific mechanisms of internalization**. (A) Assessment of the percentage of passively entered particles by measuring at 4°C. The results are depicted as increase of SSC median compared to untreated NR8383 cells. (B - D) Preincubation for 30 min with cytochalasin D (CytD), chlorpromazine (Chl) or an antibody against FcγRII (FcR ab) in order to block active uptake mechanisms, i.e. actin cytoskeleton-dependent uptake, uptake via CCP formation and FcγRII-mediated internalization, respectively. Treatment with (B) ufTiO_2 _, (C) fTiO_2 _or (D) DQ12 particles in concentrations of 10, 20 or 40 μg/cm^2 ^for 1 h shows particle-specific internalization routes. Figures represent median ± SEM as percentage of untreated or vehicle (DMSO) control cells of three independent experiments, with * p < 0.05, ** p < 0.01 and *** p < 0.001 (ANOVA with Dunnett post-hoc comparison); as well as # p < 0.05, ## p < 0.01 and ### p < 0.001 for reduced granularity vs. appropriate particle treated cells (ANOVA with LSD post-hoc comparison), respectively.

In order to investigate internalization pathways for the different particle types in NR8383 cells, various approaches were used. Passive translocation was addressed by comparative evaluation of uptake at temperatures of 37°C and 4°C. Results of these measurements are shown in Figure 9A. Irrespective of their size, approximately 50% of the TiO_2 _particles entered the cell by non-active routes, whereas for DQ12 particles a lower percentage was detected. However, it is important to note that the increased granularity of NR8383 cells at 4°C may also - at least partially - reflect a fraction of particles that was not internalized but merely adherent to the cells.

For the evaluation of specific mechanisms of active uptake a set of specific inhibitors was used. Comparison of the FACS analysis data at 1 and 3 h shows that the majority of particle uptake takes place within the first hour after particle treatment (Figure 2A-C). Hence, the particle treatment time for the inhibition experiments was set to 1 h (Figure 9B-D). Inhibition of actin-dependent phagocytosis and macropinocytosis using CytD reduced the uptake of fTiO_2 _and DQ12 particles, but not of ufTiO_2 _particles. Whereas inhibition of actin in NR8383 cells was able to abrogate active DQ12 uptake, internalization of fTiO_2 _could not be blocked completely via this mechanism. The uptake of fTiO_2 _and DQ12 particles was significantly reduced at a particle concentration ≤ 20 μg/cm^2 ^upon inhibition of the formation of CCP by Chl. Inhibition of CPP-mediated internalization of ufTiO_2 _particles was only significant at the highest concentration tested. In contrast to these findings, treatment of NR8383 cells with an antibody against the phagocytosis receptor FcγII had a strong influence on the uptake of ufTiO_2 _but not of fTiO_2 _particles. To inhibit the formation of caveolae, the inhibitor filipin III was used. However, these experiments did not demonstrate any effect on particle uptake (data not shown). Independent experiments revealed that the filipin III concentration used could reduce uptake of the fluorescent dye Alexa555-Cholera Toxin B-subunit (CTB) by the NR8383 cells. Since CTB is known to be endocytosed through lipid rafts [38,39] it is unlikely that caveolae-mediated endocytosis does play a significant role in the uptake of any of the tested particles in the NR8383 cells. No significant effects on particle uptake were observed upon treatment of the cells with the vehicle control DMSO as well as with the IgG control that was used for the FcγRII antibody treatment experiments.

## Discussion

In various studies ufTiO_2 _particles have been shown to possess increased inflammogenic potential in comparison to fTiO_2 _[2,8,40,41]. Physiologic and systemic reactions towards NP exposure, including the ufTiO_2 _that was used in this study, have been shown in several *in vivo *investigations [6,9,42,43]. However, for the investigation of underlying basic cellular mechanisms and pathways, *in vitro *studies are necessary with established cell lines, e.g. NR8383 cells [19]. Responses of these cells to various toxicants such as PMA, endotoxin and DQ12 were shown to be highly comparable to those in primary AM obtained from rat lungs by bronchoalveolar lavage [44,45].

In the present study we observed a rapid internalization of fTiO_2 _, ufTiO_2 _and DQ12 by AM in a clear dose-dependent manner. Direct comparison between the specific particles was not possible, because of intrinsic differences in their light scattering properties as observed under cell free testing conditions. However, our study demonstrates that, although all three particle types are taken up, the cellular responses of the AM are substantially different. DQ12 and ufTiO_2 _showed similar cytotoxicity, while significant effects for fTiO_2 _were absent. This confirms the accepted view in particle toxicology that uptake of inorganic poorly soluble particles does not necessarily culminate in a toxic response in AM. Our findings also show that particle uptake *per se *does not dictate oxidative stress and the induction of inflammatory mediators. DQ12 represented the most potent sample in inducing NF-κB activation and release of TNF-α and IL-1β from the AM. HO-1 and iNOS mRNA expression levels in AM were most pronounced after treatment with ufTiO_2 _. Remarkably also, both DQ12 and ufTiO_2 _triggered TNF-α release, while only DQ12 induced IL-1β release.

The contrasting abilities of DQ12, ufTiO_2 _and fTiO_2 _to induce IL-1β and/or TNF-α release can likely be explained by underlying differences in signaling pathways of activation of both inflammatory genes. NF-κB, a key regulator in the pathogenesis of particle-induced diseases [15,46], controls the expression of cytokines, growth factors and distinct enzymes in response to ligation of many receptors involved in immunity [47]. Indeed, in our current study, an association between TNF-α levels in the supernatants from AM upon particle treatment at equal mass (i.e. DQ12 > ufTiO_2 _> fTiO_2 _) and their abilities to cause NF-κB p65 nuclear translocation was found. The exclusive effect of DQ12 on IL-1β release is likely to be explained by the recently unraveled mechanism of its cellular activation via the inflammasome. IL-1β is produced as the inactive cytoplasmic precursor proIL-1β which has to be cleaved by caspase-1 to generate the mature active form of the protein [48-50]. In turn, caspase-1 is regulated by the inflammasome protein complex NALP3 [51], which has been proposed to be activated by crystalline silica particles following lysosomal rupture [52] or by NADPH oxidase-generated ROS driven by phagocytosis [53]. A recent study has revealed that upon priming with LPS (to induce proIL-1β), both DQ12 and ufTiO_2 _trigger IL-1β secretion from bone marrow derived dendritic cells from wild-type but not caspase-1 or NLRP3-deficient mice [54]. This suggests that the contrasting IL-1β responses observed with NR8383 cells may be due to differences in the abilities of specific types of poorly soluble particles to act on proIL-1β activation, i.e. upstream of the inflammasome activation.

A further remarkable observation in our study concerned the mRNA expression of HO-1 and iNOS. The positive control DQ12 appeared to be less potent than ufTiO_2 _with regard to the induction of mRNA expression of both genes. HO-1 is considered as a sensitive marker of oxidative stress and has shown to be induced by inhaled ambient ultrafine particles [55] as well as by DQ12 quartz [45,56]. The induction of iNOS in macrophages has been well-established in previous studies for crystalline silica particles, and this is considered to play a major role in its pulmonary toxicity [57]. The contrasts in nuclear translocation of NF-κBp65, iNOS and HO-1 mRNA expression in NR8383 cells in response to ufTiO_2 _and DQ12 suggest that particle-induced iNOS activation in AM can occur in an NF-κB-independent manner. Thus, while ufTiO_2 _and DQ12 both trigger pro-inflammatory effects unlike fTiO_2 _, these particles likely activate AM through different mechanisms.

In our present study, calcium influx and intracellular ROS generation were observed in AM with all three particle types to a similar extent, although both are considered as key mechanisms for adverse particle effects [58]. Tian and colleagues [59] recently demonstrated that ROS do not modulate [Ca^2+^]_i _in quartz-treated rat AM, however, calcium increase in the cytoplasm causes ROS generation after silica treatment. Enhanced [Ca^2+^]_i _in relation to pro-inflammatory signaling pathways has also been observed after treatment of macrophages with ultrafine carbon black (CB) particles in contrast to fine CB [27]. To the best of our knowledge, a comparison of effects between fTiO_2 _and ufTiO_2 _on calcium homeostasis in macrophages has not yet been performed. We observed no clear difference between both particle types in terms of the number of activated cells or the intensity of activation. In line with this, intracellular ROS levels also did not differ after treatment with both types of TiO_2 _. Our findings are in contrast to observations with CB [27] and suggest that particle size- and/or surface area-dependent effects on calcium influx and ROS formation are (nano)particle type-specific.

Besides intracellular ROS by DCFH-DA assay, we also determined extracellular ROS levels by means of EPR. Significant increases were observed after treatment with DQ12 and ufTiO_2 _, but not after fTiO_2 _. Previous studies indicate that fTiO_2 _and ufTiO_2 _samples do not markedly differ in their intrinsic ROS generating capacity, when measured in cell free assays in the absence of photosensitization [8,60,61]. In concordance with our current findings in NR8383 cells, we could previously also show that ufTiO_2 _, unlike fTiO_2 _, caused enhanced ROS formation in supernatants of A549 human lung epithelial cells. This suggests that ROS predominantly originate from interactions between ufTiO_2 _and cellular constituents and compartments rather than from the particles themselves. Potential relevant sources herein include NADPH oxidase enzyme family members as well as mitochondria [8]. Our findings indicate that different ROS-generating mechanisms exist in AM, with a selective sensitivity towards particle size or chemical composition as already concluded by Dick and colleagues [62]. At this stage however, it should be emphasized that the calcium imaging experiments and both ROS assays were not performed in complete culture medium, but in saline, or HBSS^+/+^, respectively. This was required to minimize potent radical scavenging properties of various (protein) constituents in the FCS-containing medium that can interfere with the assays. DLS measurements on unfiltered samples demonstrated that both fTiO_2 _and ufTiO_2 _, when suspended in HBSS, reside as large agglomerates with an average hydrodynamic diameter of 936.6 or 2018 nm, respectively, unlike in FCS-containing medium (see Table 1). Lacking differences in calcium influx and intracellular ROS between fTiO_2 _and ufTiO_2 _may therefore reflect an "agglomeration"-response of NR8383 cells. Interestingly though, increased extracellular ROS levels could be shown for ufTiO_2 _by EPR analysis, despite its agglomeration. All other parameters in our study were evaluated using FCS-containing culture medium, in which the number-average diameter of ufTiO_2 _sample was well within the nanosize range. However, apart from effects on agglomeration behavior, these treatment conditions also generate so-called (protein) coronas, most probably in a material specific manner [63]. This should be taken into account with regard to the various effects described in our study.

The importance of agglomeration and (protein) coating effects can be demonstrated from the comparative mRNA expression measurement of HO-1 and iNOS in HBSS treated versus full medium treated NR8383 cells (Figure 8). When suspended in HBSS, ufTiO_2 _failed to cause a significant increase of HO-1 mRNA, which indicated that the agglomeration state (see Table 1) of this sample is crucial for its ability to induce this oxidative stress marker. Remarkably however, the induction of iNOS by ufTiO_2 _was not abrogated. This suggests that the activation of HO-1 and iNOS by particles involves, at least in part, different signaling pathways driven by different physico-chemical properties. In contrast to the ultrafine TiO_2 _, the crystalline silica sample induced HO-1 and iNOS under both treatment conditions. At the lower treatment concentration (10 μg/cm^2^), the effect of DQ12 tended to be even stronger in the HBSS than in the FCS-containing medium. Current observations are in concordance with previous investigations in our laboratory where chemical coating of DQ12 was shown to abrogate its pro-inflammatory properties in NR8383 cells [45,64], as well as *in vivo *in the rat lung [32].

The goal of our study was to investigate the interactions between particles and AM and their associated pro-inflammatory effects in relation to particle size and physico-chemical properties. The contrasting cellular responses observed by the three types of particles could not be explained by uptake by the AM *per se*. Therefore, investigations were performed addressing the underlying cellular mechanisms of particle internalization. Herein, the importance of particle size and distribution as well as of agglomeration behavior in cell culture medium suspensions was taken into account. We also specifically compared the uptake mechanisms for both TiO_2 _samples with those that we previously investigated for DQ12 in NR8383 cells [20,65]. Evaluation of uptake at 4°C indicates a passive, energy-independent entrance of particles into cells and/or their adherence to outer membranes of AM. For both TiO_2 _samples the proportion was found to be higher than for DQ12. However, no clear difference could be seen between fTiO_2 _and ufTiO_2 _despite the marked differences in their size distributions in complete culture medium used for the uptake experiments. These observations are in line with previous findings for both materials concerning their uptake into A549 human lung epithelial cells [8].

A series of specific inhibition experiments were performed to investigate the various active uptake routes in NR8383 cells. A combination between different uptake mechanisms in our study can be reasoned by the findings of Rothen-Rutishauser and colleagues [66] showing TiO_2 _particles in a three-dimensional cell culture model free in the cytoplasm as well as membrane-bound. Our own findings indicate that the active internalization of ufTiO_2 _particles in AM is mainly performed via a FcγRII-mediated mechanism and, to a lesser extent, by CCP which exhibit a vesicle diameter of 100 - 120 nm [67]. Uptake of fTiO_2 _particles also took place via CCP, but in addition an actin-dependent uptake mechanism was equally involved. This may include macropinocytosis, by which large vesicles between 0.2 - 10 μm are formed spontaneously or upon stimulation [68]. Actin-mediated endocytosis is connected to receptor activation like MARCO and SR-A mediated processes, as previously shown by Kobzik and co-workers for primary AM of different species [69-71]. The prominent receptor-mediated uptake mechanism for fTiO_2 _and silica by human macrophages via SR-A reported by Thakur et al. [24] is beyond all question for our study, since NR8383 cells lack this receptor as determined by PCR analysis (data not shown). As expected from their size distribution, the DQ12 particles were taken up by actin-dependent classical phagocytosis which is described to be mediated by FcγRII [65]. Phagocytosis is the most effective clearance mechanism for particles between 1 - 5 μm in diameter [67]. Inhibition experiments with filipin III were found to be unsuccessful in reducing particle uptake in NR8383 cells. For DQ12 and fTiO_2 _this could be anticipated in view of their size distributions and the typical diameter of 50 - 100 nm of the caveolae vesicles. However, filipin III was also ineffective for ufTiO_2 _, despite the fact that its number-average hydrodynamic diameter falls into the vesicle size range of caveolae. This suggests that even for these smaller particles/aggregates alternative uptake pathways such as CCP dominate. Notably, apart from differences in primary particle size and agglomeration behavior, both TiO_2 _samples also differ in their chemical composition. Therefore, a potential role of rutile vs. anatase in particle-macrophage interactions can not be ruled out. Another explanation may be related to the specific method of particle uptake used in the present study. In relation to the relative contribution of particle number and particle mass to changes in AM granularity it is possible that the uptake of the smallest particles is underestimated in the flow cytometry approach. However, in a recent study it was demonstrated that flow cytometry can detect organosilica nanoparticles as small as 58 nm in diameter via side scattering analysis and that the method is actually suitable for size distribution analysis [72]. Comparison of the TEM distribution data of the samples used in our study with SSC histograms of cell free particle suspensions obtained with the same apparatus (FACS Calibur) indicates that this is also valid for TiO_2 _(data not shown).

A major conclusion that can be drawn from the uptake experiments is that fTiO_2 _and DQ12 which are both classified as "fine" particles show a specificity and size-dependency with regard to the tested cellular uptake mechanisms. Further studies are needed to investigate these alternative uptake pathways using independent inhibition strategies (e.g. siRNA). Because of the limitations of the cell line used in our present study (e.g. lack of SR), this should be done preferably with primary macrophages. These studies should also focus on the potential contribution of particle-specific coronas to kinetics and pathways of uptake and associated cellular responses.

Our observation that multiple uptake mechanisms may be relevant for one specific type of particle can be explained by the size-distribution of the specific samples and, likely of more importance, their agglomeration behavior when suspended in culture media. Obviously, for the identification of the *in vivo *relevant mechanisms of uptake of specific types of (nano)particles by AM, one should obviously take into account the role of the micro-environment of these cells, e.g. potential corona-forming constituents of the alveolar lining fluid. Nevertheless, our current *in vitro *findings emphasize on the aspect that the inflammatory properties are not driven by uptake *per se*. It appeared that specific physico-chemical properties of quartz, fTiO_2 _and ufTiO_2 _particles are responsible for qualitative as well as quantitative differences in their ability to induce oxidative stress and inflammatory responses in AM. In contrast to fTiO_2 _which was relatively inert, both DQ12 and ufTiO_2 _increased extracellular ROS and TNF-α release, while ufTiO_2 _predominantly enhanced iNOS mRNA expression, and DQ12 exclusively triggered IL-1β release. Our findings indicate that these dissimilar macrophage responses may be related to specific differences in uptake mechanisms, respectively involving actin cytoskeleton, CCP formation and FcγRII-mediated internalization. The findings with fTiO_2 _demonstrate that actin and CCP are not necessarily involved in pro-inflammatory cytokine release and extracellular ROS generation by macrophages after particle uptake. The data for DQ12 and ufTiO_2 _indicate a role for FcγRII in macrophage responsiveness. The contrasting activation profiles for both materials emphasize the need for further investigations, specifically regarding the inflammasome. The relative overlap of the investigated mechanisms for the various types of particles used in this study are likely explained by their size distributions. Indeed, studies with monodisperse particles are most appropriate to clarify individual mechanisms of uptake by macrophages. However, it should be emphasized that AM typically encounter size ranges of particles and their agglomerates of various types of respirable materials, including the fine and ultrafine TiO_2 _samples used in our current study.

## List of abbreviations used

AM: alveolar macrophages; ANOVA: analysis of variance; AP-1: activator protein 1; [Ca^2+^]_i _: intracellular calcium concentration; CB: carbon black; CCP: clathrin coated pits; Chl: chlorpromazine; CytD: cytochalasin D; DCFH-DA: dichlorodihydrofluorescein diacetate; DF-LSM: dark field light scattering microscopy; DLS: dynamic light scattering; DMPO: 5;5-dimethyl-1-pyrroline-N-oxide; DMSO: dimethyl sulphoxide; DQ12 quartz: Dörentruper quartz; EA: Elemental analysis; EPR: Electron Paramagnetic Resonance; FCS: fetal calf serum; FcγRII: Fcγ receptor II; FSC: forward scatter; fTiO_2 _: fine titanium dioxide; GAPDH: Glycerinaldehyd-3-phosphat-Dehydrogenase; HBSS: Hank's buffered saline solution; HO-1: heme oxygenase 1; IgG1κ: Immunoglobulin G1; kappa chain; IL-1β: interleukin 1β; iNOS: inducible nitric oxide synthase; LPS: lipopolysaccaride; MARCO: Macrophage receptor with collagenous structure; NADPH: nicotinamide adenine dinucleotide phosphate; NALP3: NACHT; LRR and PYD domains-containing protein 3; NF-κB: nuclear factor kappa B; NLRP: NOD-like receptor family; NP: nanoparticles; PBS: phosphate buffered saline; PKC: protein kinase C; PLC: Phospholipase C; PMA: phorbol 12-myristate 13-acetate; ROIs: regions of interest; ROS: reactive oxygen species; SSA: specific surface area; SSC: sideward scatter; TEM: transmission electron microscopy; TGA: thermogravimetric analysis; TNF-α: tumor necrosis factor alpha; ufTiO_2 _: ultrafine titanium dioxide; WST-1: water-soluble tetrazolium.

## Competing interests

The authors declare that they have no competing interests.

## Authors' contributions

AMS performed most of the experimental work and drafted the manuscript. JL analyzed the calcium imaging experiments. AB performed the particle characterization experiments. DvB performed the qRT-PCR experiments, PH prepared light microscopic data. FJvS was involved in the design of mRNA experiments. AMS was involved in the design and analysis of the particle characterization experiments as well as helped drafting the manuscript. CRR was involved in the design and analysis of the calcium imaging experiments as well as helped drafting the manuscript. RPFS was involved in the study design, statistical analysis and helped drafting the manuscript. CA was involved in the study design and coordination and helped drafting the manuscript. All authors read, commented on and approved the manuscript.
